# Influence of High-Volume Calcined Phosphogypsum on Mechanical Properties and Freeze–Thaw Resistance of Supersulfated Slag Cement Concrete

**DOI:** 10.3390/ma19112330

**Published:** 2026-06-01

**Authors:** Fang Deng, Guanjun Han, Kaiqin Xu, Xiqiang Jia, Dan Zheng, Ling Tao, Jun Dong, Yishun Liao

**Affiliations:** 1 School of Environmental and Biological Engineering, Wuhan Technology and Business University, Wuhan 430065, China; dengfang@wtbu.edu.cn (F.D.); zhengdan@wtbu.edu.cn (D.Z.); taoling@wtbu.edu.cn (L.T.); dongjun_2217@163.com (J.D.); 2 School of Urban Construction, Wuhan University of Science and Technology, Wuhan 430065, China; 15537510364@163.com (G.H.); x577063601@163.com (K.X.); jiaxiqiangv@163.com (X.J.); 3 Junji Environmental Technology Co., Ltd., Wuhan 430073, China; 4 Hubei Provincial Engineering Research Center of Urban Regeneration, Wuhan 430065, China

**Keywords:** phosphogypsum, phosphogypsum slag cement concrete, mechanical property, frost resistance, water absorption rate

## Abstract

**Highlights:**

- Increasing CPG content decreases the slump of concrete.
- With high CPG content, concrete strength decreases while water absorption rate increases.
- An appropriate amount of CPG enhances the relative dynamic modulus of elasticity.
- Although CPG increases the mass loss rate, it remains below 3.0%.

**Abstract:**

This study investigates the effects of high-volume calcined phosphogypsum (CPG) on the workability, mechanical properties, and frost resistance of phosphogypsum slag cement concrete (PSCC). CPG was used as a sulfate activator to prepare PSCC mixtures with CPG contents ranging from 40% to 70%. The slump, flexural and compressive strengths, water absorption, relative dynamic modulus of elasticity, and mass loss rate after freeze–thaw cycles were evaluated. The results show that the slump of fresh concrete decreases from 275 mm to 35 mm as the CPG content increases from 40% to 70%. The compressive strength decreases with increasing CPG content; however, the 90-day compressive strength still ranges from 39.9 MPa to 56.4 MPa. Even at high CPG contents, the concrete maintains moderate to relatively high strength levels. At a CPG content of 40%, the water absorption rate is 5.9%, meeting the requirement of the Chinese standard JC/T 899-2016. Freeze–thaw cycle tests indicate that a higher CPG content results in a higher mass loss rate. Nevertheless, all mixtures comply with the Chinese standard JC/T 899-2016, which limits the mass loss rate to no more than 3.0% after 50 freeze–thaw cycles for curbstones. The relative dynamic modulus of elasticity shows a trend of decreasing, then increasing, with increasing CPG content.

## 1. Introduction

Phosphogypsum (PG) is the primary industrial by-product generated during the production of phosphoric acid and phosphate fertilizers in the phosphate chemical industry. Its main component is calcium sulfate dihydrate (CaSO_4_·2H_2_O), with trace amounts of phosphorus, fluorine, organic matter, and radioactive substances [1]. On average, approximately 5 tons of PG are generated for every ton of phosphoric acid [2]. Globally, PG stockpiles have exceeded 6 billion tons, with an estimated annual increase in production of around 200 million tons. The target utilization rate of PG is 65% by 2026 in China [3]. However, the total stockpile remains substantial, with 80 million tons added annually—still significantly higher than in developed countries like Belgium and Japan [4,5]. Notably, environmental issues arising from uncovered PG stockpiles are becoming increasingly prominent. Prolonged exposure to rainfall leads to leachate infiltration and surface runoff, resulting in soil acidification, the eutrophication of water bodies, and groundwater contamination [6]. In dry, windy weather conditions or during mechanical operations, PG stockpiles are prone to dust generation, contributing to air pollution. Additionally, harmful impurities in PG—such as phosphorus, mercury, cadmium, and radioactive elements—may vaporize into the atmosphere, further exacerbating environmental pollution [7].

Therefore, to promote the utilization of PG, achieve “zero-waste city” initiatives, and advance circular economy goals, many scholars have applied it to building materials. PG has low strength and poor water resistance and thus is not used alone as a cementing material. When combined with granulated blast furnace slag and alkaline activators, its performance improves significantly [8]. This composite also has good impermeability. The slag refines the matrix pore structure, blocking harmful external ions [9,10]. Moreover, PG accelerates slag dissolution, boosting the formation of AFt and C-S-H gel in hydration products [11,12]. Based on this applied research, PG slag cement, a novel hydraulic binder, has emerged. It mainly consists of granulated blast furnace slag and PG (a sulfate activator), supplemented with a small number of alkaline activators. The materials are processed through mixing and grinding. Typically, the sulfate activator content ranges from 10% to 20%, and the resulting product is also known as supersulfated cement [13,14,15]. Unlike traditional Portland cement, PG slag cement does not require high-temperature calcination, and it contains little to no cement clinker—substantially lowering both energy consumption and carbon dioxide emissions. It also offers high compressive strength, low heat of hydration, excellent resistance to sulfate attack [16], and good mechanical properties at low temperatures [17], resulting in it being increasingly considered for engineering applications. Additionally, studies have shown that PG slag cement can effectively immobilize hazardous heavy metals such as lead and cadmium [18,19] and reduce the radioactivity of aluminum-based nuclear waste [20], further highlighting its environmental benefits. Consequently, it has emerged as a prominent topic in the field of building materials in recent years.

However, the presence of organic matter, soluble phosphorus, and fluoride impurities in untreated PG can adversely affect the performance of PG slag cement. Soluble phosphorus reacts with Ca^2+^ to form Ca_3_(PO_4_)_2_, which adheres to the PG surface, reducing the concentrations of Ca^2+^ and SO_4_^2−^ in the liquid phase and consequently slowing down the hydration reaction of the cement. This leads to the formation of coarser hydration crystals and a looser structure. Additionally, the acidic impurities in PG may prolong the setting time and hinder early strength development, severely limiting its suitability for practical engineering applications [21,22,23]. Studies have shown that calcination treatment can effectively immobilize impurities in PG and enhance its performance [24,25]. During calcination, the crystal structure of PG undergoes multiple transformations and dehydration, reducing its crystal size and thereby increasing its hydration activity [26]. Simultaneously, soluble phosphorus impurities in PG gradually transform into insoluble calcium pyrophosphate (CaP_2_O_7_), with this transformation becoming more pronounced as calcination temperature and duration increase [27]. In addition, although small amounts of residual impurities (e.g., phosphates and fluorides) may still remain after calcination, their influence is significantly weakened and mainly affects hydration kinetics rather than governing the overall performance. Gong et al. [28] prepared low-density, lightweight, and performance-stable cementitious materials using Calcined PG (CPG). These materials not only enhance the mechanical properties of cement mortar but also demonstrate promising market prospects due to their low environmental impact. Wang et al. [29] found that increasing the CPG content moderately delays cement hydration and promotes the formation of greater quantities of ultimate hydration products, thereby enhancing cement strength. Furthermore, CPG has been successfully used as a primary material for manufacturing curbstones, significantly improving compressive strength and effectively addressing water-induced cracking and softening. This enhances both the moisture resistance and durability of curbstones [30]. Meanwhile, the performance of CPG-based sand-free self-leveling mortar meets the requirements of the JC/T 1023-2021 standard “Gypsum-based Self-leveling Mortar,” demonstrating a flexural strength of 12.0 MPa and a compressive strength of 45.9 MPa at 28 d, a softening coefficient of 0.886, and a water absorption of 2.8% [31].

In summary, although calcined phosphogypsum (CPG) has shown promising potential to improve the performance of cement-based materials, systematic research on its use as a sulfate activator in phosphogypsum-slag cement systems remains limited, particularly at high replacement levels. Most studies have focused on low to moderate PG content or untreated phosphogypsum, while the behavior of high-volume CPG systems remains insufficiently understood. The present study, therefore, focuses on the use of high-volume CPG (40–70%) in a PG-slag cement system. This range is particularly relevant because it represents a condition in which the balance between sulfate supply and slag availability becomes critical, significantly influencing hydration reactions, pore structure development, and overall performance. In addition, this study provides new insights into the freeze–thaw resistance of PG-slag cement concrete by linking macroscopic performance (mass loss and relative dynamic modulus) with both chemical factors (hydration products such as AFt and C-(A)-S-H) and physical factors (workability, compaction, and pore structure). Therefore, this work aims to clarify the role of high-volume CPG in determining the mechanical properties and durability of PG-slag cement systems and to provide a more comprehensive understanding of its performance.

## 2. Experiments

### 2.1. Materials

**Figure 1:**
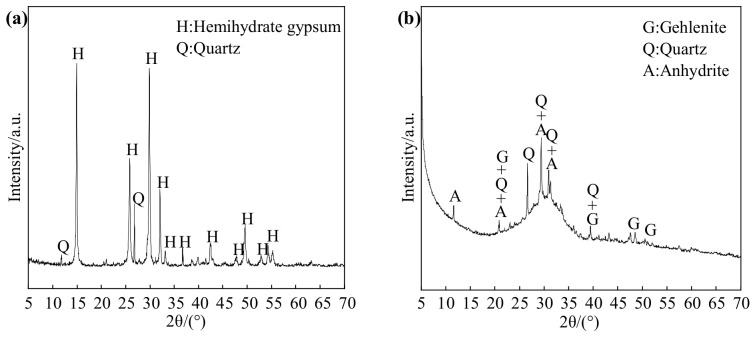
XRD patterns of raw materials (**a**) CPG and (**b**) GGBS.

CPG (pH = 5.6), sourced from Hubei Chuxing Chemical Co., Ltd. (Yichang, China), was used in the experiment. S95-grade ground granulated blast furnace slag (GGBS), supplied by Wuhan Wuxin New Building Materials Co., Ltd. (Wuhan, China) had a density of 2.9 g/cm^3^, a specific surface area of 396 m^2^/kg, and activity indices of 75.8% and 100.9% at 7 and 28 days, respectively. The chemical compositions of the raw materials are listed in Table 1, and their X-ray diffraction (XRD) patterns are shown in Figure 1. From Figure 1, it can be observed that the primary mineral components of CPG are hemihydrate gypsum and quartz. From GGBS, the main mineral components include gehlenite, anhydrite, and quartz.

The quicklime used in this study had a CaO content of 98% (obtained from Tianjin Zhonglian Chemical Reagent Co., Ltd., Tianjin, China). River sand, with a fineness modulus of 2.56, served as the fine aggregate. The coarse aggregate was crushed stone with a particle size range of 5–25 mm and a continuous gradation, with the sand ratio set at 40%. The polycarboxylate ether (PCE) superplasticizer employed had a water-reducing rate of 35% (produced by Wuhan Xingxiang Technology Co., Ltd., Wuhan, China). A protein-based gypsum retarder (manufactured by Shanghai Chenqi Chemical Technology Co., Ltd., Shanghai, China) and an organic silicon hydrophobic agent (with a solid content of 55%, provided by Beijing Jiasheng Building Materials Co., Ltd., Beijing, China) were also used. The dosages of the superplasticizer, retarder, and hydrophobic agent were 0.7%, 0.1%, and 0.25%, respectively, based on the mass of the cementitious material. Tap water was used as the mixing water.

Alkaline activators provide the necessary alkaline environment for slag dissolution and the hydration of gypsum-slag cement. However, the alkalinity of the system must be maintained at a moderate level, as excessively high or low alkalinity will both lead to destabilization of the ettringite structure [27]. Research [32] indicates that an optimal pH range of 10.8 to 12.5 is more conducive to ettringite formation. Given this and considering that PG slag cement pastes exhibit relatively high strength alongside good water resistance when the PG content is between 40% and 70% [32,33], the present study focuses on this content range for in-depth investigation.

Concrete specimens with CPG varying from 40 to 70% and a water-to-binder ratio of 0.38 were prepared for slump, strength, water absorption rate, and freeze–thaw cycle tests. Table 2 lists the proportions of the mixture for the specimens. The specimens were prepared in accordance with the Standard for Test Methods GB/T 50080-2016 [34]. First, CPG, GGBS, lime, gravel, and sand were placed into the mixer and mixed at a constant speed for 1 min. Meanwhile, the retarder, PCE, and hydrophobic agent were added to the mixing water and thoroughly blended. The admixture-containing water was then slowly and evenly poured into the mixer, and mixing continued for an additional 2 min until a uniform mixture was obtained. The fresh concrete was poured into molds and compacted on a vibrating table for 15 s. Once a slight bleed appeared on the surface, a rubber mallet was used to tap the mold to ensure proper compaction. The samples were left to cure at room temperature for 24 h before demolding and then transferred to a standard curing room (temperature: (20 ± 2) °C, relative humidity > 95%) until the specified curing age was reached.

A polycarboxylate superplasticizer (PCE) was added at 0.7% by mass of binder (5.04 kg/m^3^) to ensure adequate workability. A retarder (0.72 kg/m^3^) was incorporated to mitigate the rapid setting behavior of phosphogypsum-based systems. In addition, a hydrophobic agent (1.8 kg/m^3^) was used to reduce water ingress and improve durability performance.

The cement paste samples for SEM testing were prepared in accordance with GB/T 1346-2024 [35], using a fixed water-to-binder ratio of 0.38, the mix proportions were identical to those of the concrete, except that no coarse or fine aggregates were included. Detailed mix proportions are provided in Table 3. According to the proportions in Table 3, the admixtures—retarder, PCE, and hydrophobic agent—were first dissolved in the mixing water and stirred thoroughly. Then, the weighed binders (CPG, GGBS, and lime) were added to the mixer and dry-mixed uniformly. The prepared admixture solution was then slowly poured into the mixer. The mixing procedure was as follows: mix at low speed for 2 min, pause for 15 s (during which the paste was scraped from the mixing blades and the inner wall of the bowl into the center), then mix at high speed for another 2 min. The freshly mixed paste was immediately cast into molds and cured under standard conditions until the specified curing age. At the end of the curing period, hydration was terminated using absolute ethanol, and the samples were sealed and stored for subsequent SEM analysis.

### 2.2. Test Methods

For each test condition, at least three specimens were prepared and tested. The reported results represent the average values of the measured data. The slump test was conducted according to GB/T 50080-2016 [34]. The ambient temperature was maintained at 20 ± 5 °C. Weighing accuracy was controlled within ±1% for aggregates and ±0.5% for water and cementitious materials. Samples were taken from multiple points within the same batch to ensure representativeness, and testing began within 5 min of sampling. Before testing, the slump cone and base plate were moistened and secured. Fresh concrete was filled into the cone in three equal layers, each layer being tamped 25 times with a standard rod, with the rod penetrating into the previous layer. After leveling the top, the cone was lifted vertically in 3–7 s. Once the sample stabilized or after 30 s, the slump was measured as the difference between the height of the cone and the highest point of the slumped concrete, recorded to the nearest 5 mm. The entire process from filling to lifting the cone was completed within 150 s. If the sample collapsed or sheared, the test was repeated. Two consecutive failures indicated poor workability.

Mechanical performance tests were conducted in accordance with GB/T 50081-2019 [36]. Cube samples (100 mm × 100 mm × 100 mm) were prepared for compressive strength testing, while prismatic samples (100 mm × 100 mm × 400 mm) were used for flexural strength testing. After casting, the samples were demolded after 1 d and cured under standard curing conditions, with regular watering to maintain adequate moisture until the specified curing age, which included 3, 7, 28, 56, and 90 days. The compressive strength test was applied a continuous load at a rate of 0.5 MPa/s until sample failure, while the flexural strength test was conducted at a uniform loading rate of 0.05 MPa/s.

Water absorption tests of samples with a size of 100 mm × 100 mm × 100 mm were conducted in accordance with JC/T 899-2016 [37]. Samples cured under standard conditions for 28 d were first dried and weighed, then soaked in water for 1 d. After soaking, the samples were wiped with a wrung-out wet towel to remove surface water, then weighed again. The water absorption rate was calculated as follows:

(1) $$W=\frac{m_{1}}{m_{2}}\times 100\%$$

where W denotes the water absorption rate (%), m_0_ represents the mass of the sample before soaking (g), and m1 indicates the mass after soaking (g).

Freeze–thaw cycle tests were conducted in accordance with GB/T 50082-2024 [38]. Concrete samples measuring 100 mm × 100 mm × 400 mm were initially cured under standard conditions for 24 d. Subsequently, they were transferred to water at (20 ± 2) °C, ensuring the water level was (20–30) mm above the sample tops, and soaked for 4 d. Finally, the freeze–thaw tests were initiated at 28 d. Each freeze–thaw cycle lasted 2–4 h and included both freezing and thawing phases. During the freezing phase, the temperature was reduced from −18 to 5 °C, while in the thawing phase, it was increased from 5 to −18 °C. The transition between the two phases lasted no more than 10 min, and the thawing stage accounted for at least one-quarter of the total freeze–thaw cycle duration. After every 25 freeze–thaw cycles, the samples were removed to measure their mass loss rate and relative dynamic modulus of elasticity, and any visible surface changes were carefully recorded. The testing was concluded after 50 freeze–thaw cycles.

The mass of the samples was measured using an electronic balance with an accuracy of 1 g. The mass loss rate of the samples after the freeze–thaw cycles was calculated as follows:

(2) $${\Delta W}_{n}={(W}_{0}-W_{n}{)/W}_{0}\times 100\%$$

where ΔW_n_ is the mass loss rate after n freeze–thaw cycles (%), W_0_ denotes the initial mass of the samples (kg), and W_n_ indicates the mass after n freeze–thaw cycles (kg).

The transverse fundamental frequency of the concrete samples was measured using the DT-16 dynamic modulus testing instrument. The relative dynamic modulus of elasticity of the sample is calculated as follows:

(3) $$P_{n}=f_{n}^{2}/f_{0}^{2}\times 100\%$$

where P_n_ denotes the relative dynamic elastic modulus after n freeze–thaw cycles (%), f_0_ indicates the initial transverse fundamental frequency of the sample (Hz), and f_n_ represents the transverse fundamental frequency after n freeze–thaw cycles (Hz).

The XRD results discussed in this study were obtained from our previous work on the same CPG-slag cement paste system [39]. Since the paste compositions are identical to those used in the present study (except for the absence of aggregates), the previously published XRD data are cited here to support the interpretation of GSCC’s macroscopic performance.

Samples cured for 3 and 28 d were examined using a TESCAN MIRA LMS scanning electron microscope (TESCAN GROUP, a.s., Brno, Czech Republic). Cement paste samples were cured at (20 ± 1) °C until the designated ages. After curing, samples were fractured to extract material from the uncarbonated core region. Hydration was terminated by immersion in anhydrous ethanol for 24 h, followed by vacuum drying at 40 °C for at least 72 h. For SEM analysis, samples with both diameter and thickness not exceeding 10 mm were prepared. The testing surface was required to be a natural fracture surface and relatively flat.

To broaden the normative framework of the experimental evaluation, the Chinese standards adopted in this study were compared with relevant international standards. For the test of freeze–thaw resistance, the Chinese standard GB/T 50082-2024 is comparable to ASTM C666/C666M [40] and EN 12390-9 [41], although the exact cycling regime, specimen geometry, and termination criteria differ among standards. In addition, since the studied material has potential application in concrete curbs, EN 1340 [42] was considered as an international reference for the water absorption, flexural strength, and freeze–thaw durability of concrete curb units.

## 3. Results and Discussion

### 3.1. Physical Properties

#### 3.1.1. Workability

**Figure 2:**
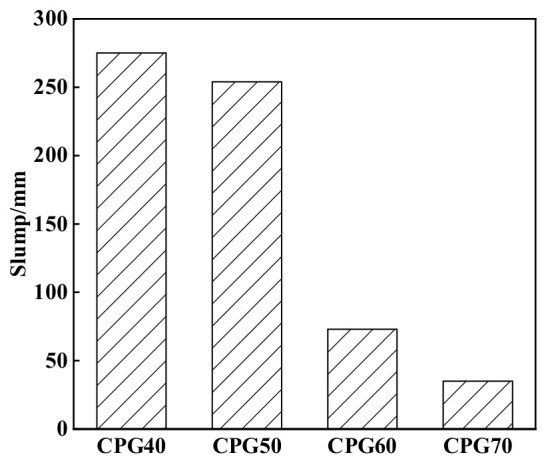
Slump of fresh concrete with different contents of CPG.

The slump of fresh concrete with different CPG contents is shown in Figure 2. As illustrated, the slump of the concrete decreases with the increase in the CPG content. Specifically, when the CPG content increases from 40% to 70%, the slump of concrete decreases from 275 to 35 mm—a reduction of 87.3%. This trend may be attributed to two main factors: firstly, hemihydrate gypsum undergoes rapid hydration upon contact with water, forming dihydrate gypsum:

(4) $${CaSO}_{4}\cdot {0.5H}_{2}O+{1.5H}_{2}O\to {CaSO}_{4}\cdot {2H}_{2}O$$

This reaction is likely to consume a large amount of free water in the concrete but also results in the formation of a three-dimensional network flocculation structure. This structure may increase interparticle friction resistance within the paste, thereby reducing its fluidity. At higher CPG contents, particularly beyond 60%, undispersed gypsum particles tend to agglomerate, further aggravating slump loss [15]. Secondly, increasing CPG content reduces the relative amount of slag, weakening its beneficial effect on rheological properties. Under alkaline conditions, slag depolymerization releases [SiO_4_]^4−^ and [AlO_4_]^5−^ species that improve fluidity [43]; however, this effect diminishes as slag content decreases. In addition, the evolution of alkalinity in the phosphogypsum-slag system influences early hydration behavior [44,45]. However, this effect is strongly dependent on slag content. When the CPG content is below approximately 50%, sufficient slag helps maintain paste rheology. In contrast, once the CPG content exceeds this level, the reduced slag content limits this effect, and the system becomes dominated by gypsum-related behavior. Specifically, the increased gypsum content leads to higher water demand, stronger particle interactions, and reduced dispersing efficiency of superplasticizers, as reported in sulfate-rich systems [15,43]. As CPG content continues to exceed this critical range, the combined effects of water consumption, slag reduction, and particle agglomeration lead to a sharp increase in interparticle friction, resulting in an abrupt drop in slump. Furthermore, the severe loss of workability at high CPG contents may impair compaction, leading to increased entrapped air and internal porosity. These physical defects weaken the paste–aggregate interface, contributing to the observed reduction in strength and durability.

#### 3.1.2. Mechanical Properties

**Figure 3:**
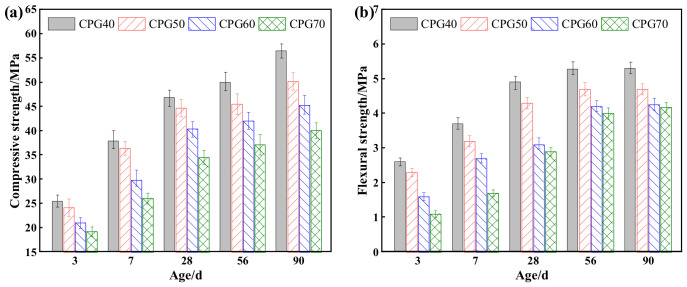
Strength of concrete with different contents of CPG: (**a**) compressive strength and (**b**) flexural strength.

The strength of concrete with different CPG contents is shown in Figure 3. Notably, as the curing age increases, both these strengths improve.

As shown in Figure 3a, the changes in compressive strength follow a trend similar to that of flexural strength, gradually decreasing with increasing CPG content. At 3 d, the compressive strength is 25.4 MPa with a CPG content of 40%. As the CPG content increases from 40% to 70%, the compressive strength decreases to 19.3 MPa, representing a 24.0% reduction. At 28 d, the compressive strength reaches 34.5 MPa with 70% CPG content, which is 26.4% lower than the 46.9 MPa observed at 40% CPG content. As hydration progresses, the compressive strength development slows down after 56 d. However, by 90 d, the compressive strength of the samples with 40% CPG increases to 56.4 MPa, which is 41.3% higher than that of the samples with 70% CPG content.

As shown in Figure 3b, the flexural strength of the concrete decreases gradually with increasing CPG content at all curing ages. At 3 d, with a CPG content of 40%, the flexural strength of the concrete is 2.6 MPa. When the CPG content increases from 40% to 70%, the flexural strength decreases to 1.1 MPa—a reduction of 57.7%. At 28 d, with 40% CPG content, the flexural strength reaches 4.9 MPa. As hydration continues, the flexural strength stabilizes after 56 d. By 90 d, at 40% CPG content, the flexural strength reaches its maximum value of 5.3 MPa, representing a 26.2% increase compared to the 4.2 MPa observed at 70% CPG content. This trend is consistent with previous studies, which reported that excessive sulfate or gypsum accumulation may reduce the amount of effective cementitious products and weaken the mechanical properties of concrete [46].

The strength of the hardened matrix is generally considered to be controlled by the formation of hydration products and the resulting pore structure. The generated AFt interlocks with gypsum dihydrate to form a skeletal framework and, together with C-(A)-S-H gel, binds to create a dense three-dimensional spatial structure. This refinement of the pore structure may contribute to a more compact hardened matrix, thereby continuously increasing the concrete’s strength. Under alkaline conditions, the slag powder gradually dissolves and reacts with gypsum dihydrate, forming both AFt and C-(A)-S-H gel. These hydration products tend to fill the pores among unhydrated gypsum dihydrate crystals, refining the pore structure of the hardened matrix [47]. However, at high CPG contents, the reduced slag proportion limits the formation of hydration products. This may lead to an insufficiency of hydration products to fully fill the pore structure of the paste, thus inhibiting the strength development of the concrete. In addition to reduced hydration products, decreased workability at high CPG contents may lead to insufficient compaction and increased air void content, further contributing to strength deterioration.

Within the investigated system, the 40% CPG (CPG40) mixture can be regarded as a reference composition, as it consistently exhibits the highest strength and better overall performance. Therefore, the observed trends primarily reflect the relative influence of increasing CPG content rather than an absolute comparison with conventional cement systems.

#### 3.1.3. Water Resistance

**Figure 4:**
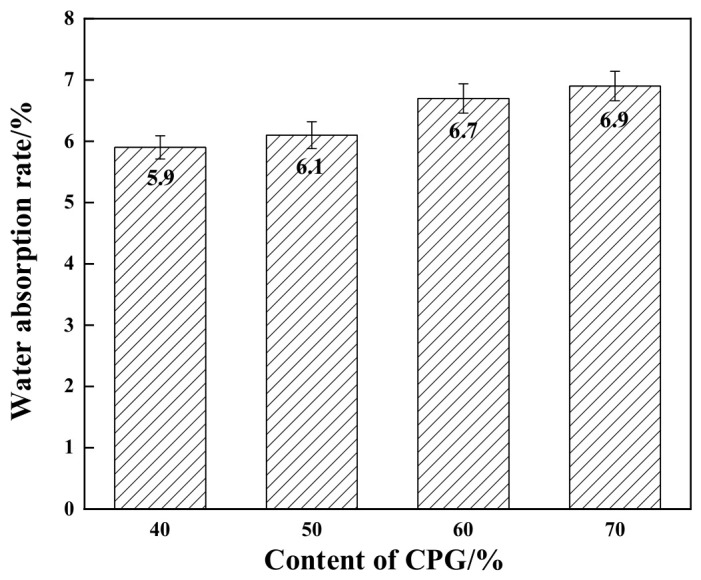
Water absorption of concrete with different contents of CPG.

The water absorption of concrete with different CPG contents is shown in Figure 4. As illustrated, the water absorption rate gradually increases with high CPG content. When the CPG content exceeds 50%, the water absorption rate exceeds 6%. At 40% CPG content, the water absorption rate is 5.9%, meeting the JC/T 899-2016 “Concrete curbs” requirement of not exceeding 6.0%. At 50% CPG content, the water absorption rate increases to 6.1%; at 70% CPG content, it reaches a maximum of 6.9%. Similar results have been reported in previous studies, where increased pore connectivity and pore size significantly enhance water transport and reduce durability performance under freeze–thaw conditions [48,49].

In an alkaline environment, slag gradually dissolves and reacts with gypsum dihydrate to form AFt and C-(A)-S-H gel. These products effectively fill in the pores among unhydrated gypsum dihydrate crystals, thereby refining the pore structure of the hardened matrix and forming a barrier that impedes water penetration [43]. However, the gypsum dihydrate crystals formed during CPG hydration form a relatively loose, interwoven skeleton structure with numerous pores. This structural characteristic adversely affects matrix density, preventing it from achieving ideal compactness [50]. As the CPG content increases from 40% to 70%, the slag content in the paste gradually decreases, leading to a corresponding reduction in the formation of AFt and C-(A)-S-H gel as hydration products. This reduction may make the hardened matrix more permeable, providing additional pathways for water infiltration and significantly increasing the concrete’s water absorption rate. Moreover, the reduced workability at high CPG contents may lead to insufficient compaction and increased connectivity of capillary pores, providing additional pathways for water ingress and thereby increasing the water absorption rate.

The water absorption of CPG40 also indicates a moderate level of compactness for a gypsum-slag-based concrete. Although the acceptance limits in JC/T 899-2016 are directly applicable to Chinese concrete curbs, EN 1340 also uses water absorption as an important durability-related index for concrete curb units. Therefore, the water absorption of 5.9% for CPG40 suggests that this mixture is the most promising among the investigated series for curb applications. However, absorption values above 6.0% for mixtures with CPG contents of 50–70% indicate that excessive CPG may reduce the material’s competitiveness when assessed under broader durability expectations.

### 3.2. Frost Resistance

#### 3.2.1. Relative Dynamic Elastic Modulus

**Figure 5:**
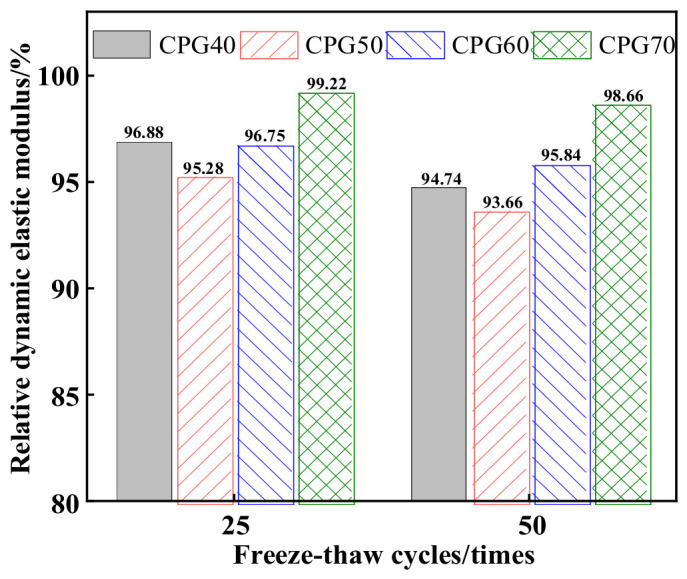
Relative dynamic elastic modulus of concrete with different contents of CPG.

The relative dynamic modulus of elasticity is a critical indicator of the frost resistance of concrete, as it effectively reflects the extent of internal damage after freeze–thaw cycles [51]. A low relative dynamic modulus of elasticity indicates severe freeze–thaw internal degradation within the sample. The relative dynamic elastic modulus of concrete with different CPG contents is shown in Figure 5. As shown, the modulus of all samples gradually decreases with increasing freeze–thaw cycles, primarily due to the inherently porous nature of concrete. According to the theory of hydrostatic pressure [52]. As the temperature decreases, the external pore solution in the concrete freezes first. The expansion of ice volume generates pressure that forces the unfrozen solution to migrate inward, thereby increasing internal pore-water pressure. After multiple freeze–thaw cycles, microcracks rapidly form and coalesce in the aggregate interfacial transition zone [53], exacerbating internal damage and substantially reducing the relative dynamic modulus of elasticity.

When the CPG content increases from 40% to 70%, the relative dynamic modulus under the same freeze–thaw cycles exhibits a trend of initially decreasing and then slightly increasing. However, this fluctuation does not necessarily indicate a real improvement in freeze–thaw resistance. At moderate CPG contents, a reduction in slag proportion leads to fewer hydration products, resulting in a less compact microstructure that allows easier water ingress. This may increase the susceptibility of the material to freeze–thaw damage and contribute to the reduction in dynamic modulus.

At higher CPG contents, although a slight increase in the relative dynamic modulus is observed, this may be associated with temporary or apparent effects, such as partial pore filling by ice or unreacted gypsum and redistribution of internal stresses during freeze–thaw cycles. These effects do not represent genuine microstructural densification and should be interpreted with caution. From a microstructural perspective, the continued reduction in hydration products and the increased formation of gypsum generally lead to a more porous and less cohesive matrix.

The freeze–thaw resistance of PG-slag cement concrete is closely related to its pore structure and hydration products. A denser microstructure with fewer connected capillary pores can effectively limit water ingress, thereby reducing internal frost damage during freeze–thaw cycles [54]. In contrast, a reduction in hydration products results in a more porous structure, facilitating water penetration and increasing susceptibility to freeze–thaw deterioration.

At higher CPG contents, the increased gypsum formation plays an important role in determining the microstructure. Gypsum crystallization and sulfate-induced expansive stresses may accelerate pore structure deterioration during freeze–thaw cycles [55]. In addition, gypsum crystals typically exhibit a plate-like morphology and relatively weak binding capacity compared with C-(A)-S-H gel. As a result, the matrix tends to form a more open, less cohesive structure, increasing pore connectivity and negatively affecting freeze–thaw resistance.

It should be noted that, although ice formation within pores may temporarily occupy void spaces under freezing conditions, this does not represent true densification of the microstructure. In general, freeze–thaw cycles generate internal stresses due to ice expansion, leading to microcrack initiation and propagation. Therefore, freeze–thaw action is more appropriately associated with pore coarsening and progressive structural deterioration than with improvement [56]. Furthermore, excessive CPG replacement may further reduce the amount of hydration products and accelerate pore structure deterioration, thereby weakening freeze–thaw resistance [57].

#### 3.2.2. Mass Loss Rate

**Figure 6:**
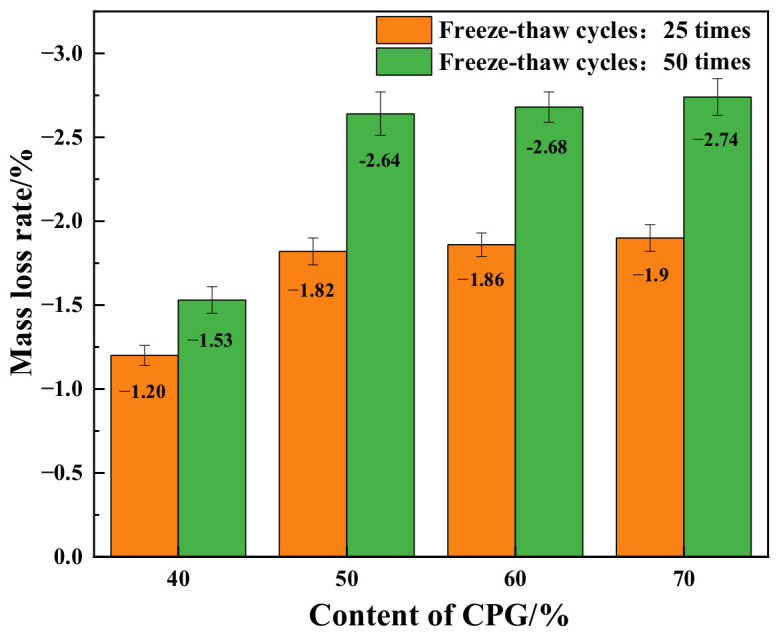
Mass loss rate of concrete with different contents of CPG.

**Figure 7:**
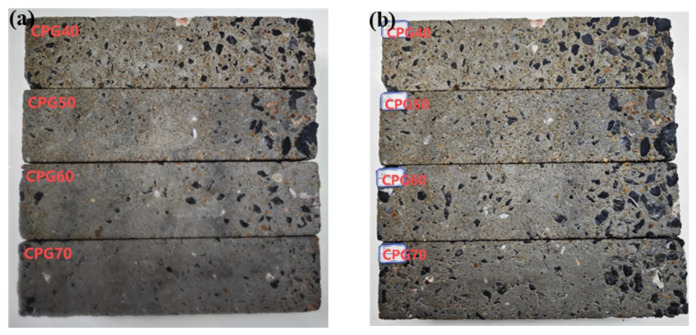
Surface condition of concrete with different contents of CPG: (**a**) 25 cycles and (**b**) 50 cycles.

Freeze–thaw cycles can cause surface spalling in concrete due to freeze–thaw expansion. This deterioration is reflected by the mass loss rate, which serves as a reliable indicator of the extent of spalling in the sample. The mass loss rate of concrete with different CPG contents is shown in Figure 6. The surface condition of concrete with different CPG contents is shown in Figure 7. From Figure 6, the mass loss rate of the samples generally increases with the number of freeze–thaw cycles. This trend is primarily due to the freeze–thaw action, which exacerbates the expansion of inherent defects—such as pores and microcracks—that inevitably form within the sample during the casting process. As water continuously infiltrates and repeatedly freezes and expands at low temperatures, the microcracks within the concrete gradually extend and connect. This process results in a progressive loss of structural integrity [58]. The surface mortar is particularly susceptible to peeling, significantly reducing the overall quality of the sample. Under the same number of freeze–thaw cycles, the mass loss rate of the samples increases with high CPG content. The mass loss rates for CPG contents of 50%, 60%, and 70% are similar. When the number of freeze–thaw cycles reaches 50, the mass loss rates for CPG-40, CPG-50, CPG-60, and CPG-70 are 1.53%, 2.64%, 2.68%, and 2.74%. All values meet the requirement specified in JC/T 899-2016 “Concrete curbs,” which stipulates that the mass loss rate after 50 freeze–thaw cycles should not exceed 3.0% in cold and severely cold regions. These results suggest that a high CPG content (>50%) may reduce concrete durability, making it more susceptible to freeze–thaw damage. This observation agrees well with previous studies demonstrating that freeze–thaw deterioration is strongly governed by pore structure evolution, microcrack propagation, and the internal stress generated by ice expansion [59].

As the CPG content and the number of freeze–thaw cycles increase, surface damage to the samples gradually worsens. This may be attributed to the continued dissolution of slag powder in an alkaline environment, where it reacts with gypsum dihydrate to form hydration products such as AFt and C-(A)-S-H gel. These hydration products tend to fill the pores among unhydrated gypsum dihydrate crystals, refining the pore structure of the hardened matrix. However, when the CPG content is high, the relative proportion of slag powder decreases. This results in a few hydration products forming at the interface transition zone, increasing the connectivity of surface pores. These open pores accelerate water penetration and freeze–thaw damage, weakening the bond between the cement paste and the aggregate. Consequently, samples with high CPG content exhibit large mass loss [60]. This result also aligns with the visual trends observed in Figure 7, which show that surface spalling becomes more severe as CPG content increases after freeze–thaw cycles. Furthermore, poor workability may lead to increased internal defects and weaker interfacial transition zones, thereby accelerating crack propagation and damage accumulation during freeze–thaw cycles.

The freeze–thaw results were further interpreted in light of international test frameworks. ASTM C666/C666M evaluates the resistance of concrete to rapid freezing and thawing, commonly by monitoring changes in the dynamic modulus and specimen deterioration, while EN 12390-9 assesses the freeze–thaw resistance of concrete using surface scaling or related durability indicators. Although the present test procedure follows GB/T 50082-2024 and is therefore not numerically identical to ASTM or EN procedures, the use of relative dynamic modulus and mass loss provides comparable information on internal damage and surface deterioration. After 50 cycles, all mixtures showed mass losses below 3.0%, with CPG40 exhibiting the lowest at 1.53%, indicating acceptable frost resistance under the adopted test regime. It should be noted that the present evaluation is limited to 50 freeze–thaw cycles in accordance with JC/T 899-2016 and thus primarily reflects performance under moderate exposure conditions rather than severe freeze–thaw environments. Nevertheless, the increasing mass loss at higher CPG contents suggests that mixtures containing more than 50% CPG may be less competitive under stricter international freeze–thaw durability requirements, especially where de-icing salts or severe exposure classes are considered.

It should be noted that the frost resistance results are interpreted relative to the CPG-slag cement system due to the absence of a conventional control mixture.

### 3.3. Hydration Products

#### 3.3.1. XRD Analysis

**Figure 8:**
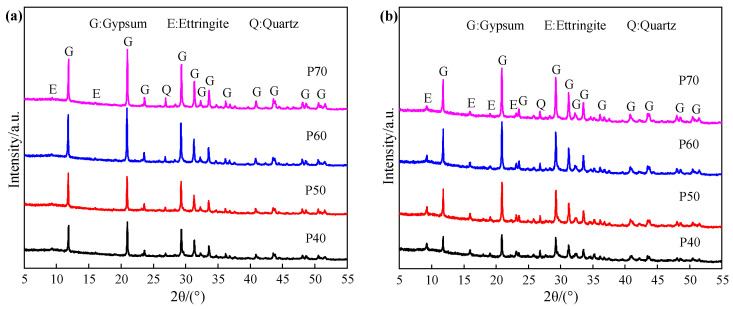
XRD pattern analysis of cement paste: (**a**) 3 d and (**b**) 28 d [39].

Figure 8 presents the XRD patterns of the samples at different ages (the corresponding semi-quantitative phase proportions are listed in Table 4). It should be noted that all XRD data presented in this section was previously reported in our earlier publication [39]. In the present study, these data are reused to support the analysis of hydration products and to provide a microstructural basis for interpreting the mechanical and durability results. The original contribution of this study lies in the systematic investigation of freeze–thaw resistance and its relationship with CPG content, which was not previously addressed. Therefore, the reuse of XRD results is scientifically justified, as it enables a more comprehensive discussion by linking microstructural characteristics with macroscopic performance. The degree of overlap with the previous publication is limited to the XRD characterization, while all experimental results related to workability, mechanical properties, water absorption, and freeze–thaw behavior are newly obtained in this study.

At early ages, the diffraction peak intensity of AFt is lower than that of gypsum dihydrate for all samples, indicating that the hydration process is initially dominated by gypsum formation. Moreover, the intensity of the gypsum diffraction peak increases with increasing CPG content. At 28 d, the previously reported results show that the gypsum diffraction peak intensity decreases compared to that at 3 d, while distinct AFt peaks appear. However, as CPG content increases, the intensity of the AFt characteristic peaks gradually decreases, whereas that of gypsum increases. This trend suggests that during hydration, gypsum dihydrate continues to participate in reactions leading to the formation of AFt, accompanied by the consumption of OH^−^ ions and a gradual reduction in the pH of the pore solution. As the CPG content increases, the formation of AFt is progressively inhibited, as reflected in the reduced diffraction peak intensity. The quantitative XRD analysis is reported in [39]. Table 4 further confirms that with increasing CPG content, the amount of gypsum dihydrate increases, while the AFt content decreases. Specifically, as the CPG content increases from 40% to 70%, the AFt content decreases significantly at both 3 d and 28 d. This reduction in AFt formation is likely associated with the observed decline in concrete strength at higher CPG contents in the present study. In addition, reduced hydration product formation weakens the interfacial bonding between the cement paste and aggregates. Consequently, during freeze–thaw cycles, samples with high CPG content exhibit greater mass loss.

#### 3.3.2. SEM Analysis

**Figure 9:**
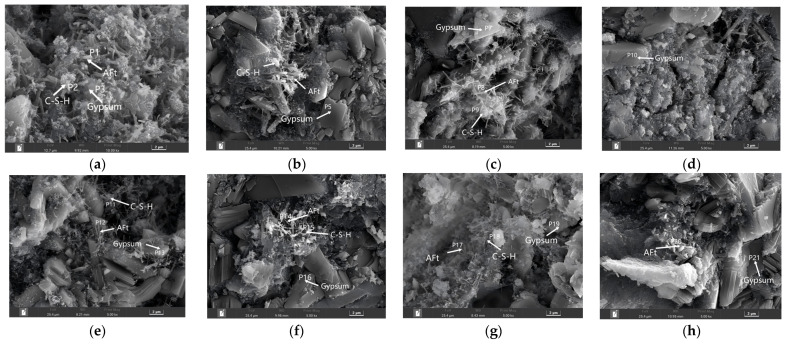
SEM images of cement paste at 3 and 28 d: (**a**) P40-3d, (**b**) P40-28d, (**c**) P50-3d, (**d**) P50-28d, (**e**) P60-3d, (**f**) P60-28d, (**g**) P70-3d, and (**h**) P70-28d.

The SEM images of CPG-slag cement pastes at curing ages of 3 and 28 d are presented in Figure 9. Elemental analysis was performed on the 21 marked points in Figure 9, and the results are shown in Table 5. As observed, the hardened paste at both curing ages mainly consists of AFt, gypsum, and C-(A)-S-H gels, whose relative proportions vary with curing time and CPG content. It is well established that the early formation of AFt contributes to the development of mechanical strength in cement-based materials. Under alkaline conditions, slag gradually depolymerizes and reacts with dihydrate gypsum to form AFt and C-(A)-S-H gels. The accumulation of these hydration products promotes the intergrowth of AFt crystals with unhydrated or partially reacted gypsum, forming an initial structural framework. This framework is further bonded and filled by C-(A)-S-H gels, resulting in a relatively dense three-dimensional network [27], which enhances the compactness of the pore structure and improves mechanical performance.

As shown in Figure 9a–c, P1, P4, and P6 exhibit needle-like AFt crystals; P2, P5, and P7 display flocculent C-(A)-S-H gel; and P3 and P8 show plate-like gypsum. These components are interconnected, forming a relatively compact microstructure. A similar structural feature is observed in Figure 9e–h. However, with increasing CPG content (e.g., P60 and P70), the amount of needle-like AFt and flocculent C-(A)-S-H gel decreases, while the proportion of plate-like gypsum increases significantly. This change is attributed to a reduction in slag content, which limits the formation of cementitious hydration products despite the higher sulfate availability. Consequently, the microstructure gradually transitions from a dense, gel-dominated system to a more porous, less cohesive gypsum-influenced structure. This observation is consistent with the XRD results reported previously [39]. From a performance perspective, this microstructural evolution provides a direct explanation for the observed reduction in mechanical strength and freeze–thaw resistance at higher CPG contents. The decrease in C-(A)-S-H gel may reduce the binding capacity of the matrix, while the increasing presence of gypsum exhibits relatively weak cohesion and plate-like morphology. Hence, it is likely to contribute to higher pore connectivity and increased water ingress. Furthermore, a clear evolution of AFt morphology with curing age can be observed. As shown in Figure 9a,c, at 3 d, AFt crystals are fine, short, and loosely distributed. In contrast, Figure 9b,f at 28 d shows that these crystals become coarser, longer, and more interconnected. This indicates the continued growth and reorganization of hydration products, which contribute to matrix densification at early stages but may also lead to increased internal stress if excessive crystallization occurs.

## 4. Conclusions

This study evaluated the effects of high-volume calcined phosphogypsum (CPG) on the mechanical properties and freeze–thaw resistance of phosphogypsum slag cement concrete (PSCC). The results demonstrate that PSCC performance is governed by the balance between sulfate supply and slag reactivity. At moderate CPG contents, the formation of AFt and C-(A)-S-H gel contributes to a relatively dense microstructure, whereas excessive CPG reduces slag availability, promotes gypsum accumulation, and results in a more porous and less cohesive matrix. The associated loss of workability at high CPG contents further aggravates compaction defects and performance deterioration.

Considering strength, water resistance, and frost durability, a CPG content of approximately 40% provides the most favorable overall performance within the investigated range. In contrast, mixtures with CPG contents above 50% exhibit reduced strength and durability, indicating that excessive replacement is not suitable for practical use. However, even at high CPG contents, the compressive strength remains above 35 MPa, indicating that the material still has potential for non-structural applications.

From an application perspective, PSCC with moderate CPG content shows potential for use in concrete curbs, pavement materials, and other precast components, contributing to both sustainable waste utilization and low-carbon construction.

This study was subject to certain limitations. In particular, the absence of a conventional OPC control mixture limited direct comparison with traditional cement systems, potentially limiting the generalizability of the findings. In addition, the freeze–thaw evaluation was limited to 50 cycles, representing moderate exposure conditions. Future work should incorporate OPC-based reference systems and extended durability testing to provide a more comprehensive assessment of the long-term performance and engineering applicability of CPG-slag cement systems.

## Figures and Tables

**Table 1:** Chemical composition of the raw materials (wt.%).

Material	CaO	SiO_2_	Al_2_O_3_	MgO	SO_3_	TiO_2_	Fe_2_O_3_	Na_2_O	K_2_O	P_2_O_5_	F^−^
CPG	43.03	9.51	0.99	0.66	36.35	-	0.40	0.17	0.29	1.91	0.86
GGBS	43.61	29.56	14.40	6.34	2.95	0.85	0.40	0.42	0.58	0.01	-

**Table 2:** Mix proportions of PG slag cement concrete (kg/m^3^).

Sample	CPG	GGBS	Lime	Gravel	Sand	Water	Retarder	PCE	Hydrophobic Agent
CPG40	288	417.6	14.4	843.84	562.56	273.6	0.72	5.04	1.8
CPG50	360	345.6	14.4	843.84	562.56	273.6	0.72	5.04	1.8
CPG60	432	273.6	14.4	843.84	562.56	273.6	0.72	5.04	1.8
CPG70	504	201.6	14.4	843.84	562.56	273.6	0.72	5.04	1.8

**Table 3:** Mix proportions of PG slag cement paste.

Sample	Mass Fraction/wt.%						W/B
	CPG	GGBS	Lime	Retarder	PCE	Hydrophobic Agent	
P40	40.00	58.00	2.00	0.10	0.70	0.25	0.38
P50	50.00	48.00	2.00	0.10	0.70	0.25	0.38
P60	60.00	38.00	2.00	0.10	0.70	0.25	0.38
P70	70.00	28.00	2.00	0.10	0.70	0.25	0.38

**Table 4:** XRD quantitative analysis of main hydration products of samples at 3 and 28 days (%) [39].

Sample	3 d			28 d		
	Ettringite	Gypsum	Quartz	Ettringite	Gypsum	Quartz
P40	18.2	74.7	7.1	17.2	77.6	5.2
P50	16.1	75.5	8.4	15.9	80.0	4.1
P60	15.2	78.3	6.5	8.6	82.4	9.0
P70	9.8	82.6	7.6	7.3	84.4	8.3

**Table 5:** Element composition analysis of hardened paste (wt.%).

Point	O	S	Ca	Al	Si
P1	40.77	10.12	39.2	4.18	5.73
P2	53.18	10.98	26.62	2.24	6.98
P3	52.93	16.58	30.49	-	-
P4	34.28	14.33	42.82	2.03	6.54
P5	44.96	21.23	33.81	-	-
P6	41.08	19.56	33.59	2.72	3.05
P7	23.77	28.32	47.92	-	-
P8	41.08	11.56	40.59	2.72	4.05
P9	53.18	10.98	26.62	2.24	6.98
P10	54.23	12.34	33.43	-	-
P11	52.87	14.90	26.29	2.35	3.59
P12	46.03	12.02	34.25	3.54	4.17
P13	44.51	20.72	34.78	-	-
P14	49.01	11.91	28.48	2.97	7.63
P15	43.66	13.85	33.82	5.03	3.64
P16	45.54	25.31	29.15	-	-
P17	45.24	11.46	35.87	4.58	2.85
P18	47.33	10.19	30.24	2.77	9.47
P19	42.16	20.02	37.82	-	-
P20	42.01	11.91	35.48	2.97	7.63
P21	34.79	24.76	40.44	-	-

## Data Availability

The original contributions presented in this study are included in the article. Further inquiries can be directed to the corresponding author.
